# A Temperature-Dependent Battery Model for Wireless Sensor Networks

**DOI:** 10.3390/s17020422

**Published:** 2017-02-22

**Authors:** Leonardo M. Rodrigues, Carlos Montez, Ricardo Moraes, Paulo Portugal, Francisco Vasques

**Affiliations:** 1 Department of Automation and Systems, UFSC—Federal University of Santa Catarina, 88040-900 Florianópolis, Brazil; carlos.montez@ufsc.br; 2 Department of Computing, UFSC—Federal University of Santa Catarina, 88905-120 Araranguá, Brazil; ricardo.moraes@ufsc.br; 3 INEGI/INESC-TEC—Faculty of Engineering, University of Porto, 4200-465 Porto, Portugal; pportugal@fe.up.pt (P.P.); vasques@fe.up.pt (F.V.)

**Keywords:** WSN, battery modeling, KiBaM, thermal effect

## Abstract

Energy consumption is a major issue in Wireless Sensor Networks (WSNs), as nodes are powered by chemical batteries with an upper bounded lifetime. Estimating the lifetime of batteries is a difficult task, as it depends on several factors, such as operating temperatures and discharge rates. Analytical battery models can be used for estimating both the battery lifetime and the voltage behavior over time. Still, available models usually do not consider the impact of operating temperatures on the battery behavior. The target of this work is to extend the widely-used Kinetic Battery Model (KiBaM) to include the effect of temperature on the battery behavior. The proposed Temperature-Dependent KiBaM (T-KiBaM) is able to handle operating temperatures, providing better estimates for the battery lifetime and voltage behavior. The performed experimental validation shows that T-KiBaM achieves an average accuracy error smaller than 0.33%, when estimating the lifetime of Ni-MH batteries for different temperature conditions. In addition, T-KiBaM significantly improves the original KiBaM voltage model. The proposed model can be easily adapted to handle other battery technologies, enabling the consideration of different WSN deployments.

## 1. Introduction

Wireless Sensor Networks (WSNs) are being increasingly used in industrial, commercial and residential facilities to support monitoring applications. The utilization of WSNs has significant restrictions, as nodes are battery-powered, and battery replacements usually have high cost operations. Therefore, it is desirable that WSN nodes use both low-power hardware components and energy-aware protocols and algorithms to save energy.

As WSN nodes can be deployed in outdoor environments or at locations subject to high temperature variations [1], the thermal effect on both the battery lifetime and its voltage behavior must be considered. The thermal effect may accelerate the rate of chemical reactions inside the battery, according to the operating temperature, implying that the battery will be able to provide higher effective capacity at higher temperatures [2]. However, using batteries at higher temperatures may present safety risks or even reduce its life cycle, i.e., its number of charge/discharge cycles [3,4]. Therefore, it is a difficult task to estimate the battery behavior at different temperatures, increasing the complexity of estimating its lifetime for typical operating scenarios.

Traditionally, WSN designers use simulators to assess the network behavior prior to its deployment. Such simulators implement battery models to emulate the behavior of nodes’ batteries [5,6], allowing the estimation of the battery lifetime [7,8,9,10,11,12,13]. The use of battery models can save both time on experimental assessments and/or investment on expensive prototypes [14]. Unfortunately, most of the available battery models do not consider the impact of operating temperature on the battery behavior [15,16], which may lead to inaccurate results [17].

The Kinetic Battery Model (KiBaM) [18] has been proposed to model the behavior of batteries that can be typically used to power WSN nodes [19]. However, it does not consider the influence of thermal effects on the battery behavior. Nevertheless, the following reasons justify its broad use: (i) it requires just the use of three parameters for modeling the battery behavior; and (ii) it presents good accuracy regarding the battery lifetime estimation, with average relative error between 2% and 4% [20,21]. Despite these accuracy values, an average relative error of 2%–4% may become relevant for typical WSN applications, where nodes can operate during long periods of time, and the estimation of its lifetime behavior is a crucial issue. Moreover, operating batteries at different temperatures may also increase the error of the battery lifetime estimation [17]. A relevant shortcoming of the original KiBaM is that it presents a linear voltage model to represent the discharge of the battery over time. This linear representation can induce a significant error on the battery lifetime estimation, particularly when considering different voltage levels as voltage cut-off points for the WSN nodes.

The target of this paper is to extend the original KiBaM [18,22], enabling the consideration of thermal effects. This extended model, called T-KiBaM, includes the concepts of chemical kinetics (modeled by an Arrhenius equation) to model the influence of temperature on the chemical reactions that occur inside the batteries. An experimental assessment performed for different operating temperatures shows that the proposed T-KiBaM estimates the behavior of Ni-MH batteries with higher accuracy, whatever the specific operating temperature, as long as the battery is kept under its nominal operating temperature range (−10–45 °C). The major contributions of this paper can be summarized as follows:

- An extension of the original KiBaM, to include the impact of thermal effects on its voltage behavior over time, enabling an improved battery lifetime estimation.
- A methodology, referred to as the Temperature-Dependent Voltage Model (TVM), that enables an accurate representation of the battery voltage behavior over time, which is highly non-linear. This methodology also enables the adjustment of the T-KiBaM voltage parameters according to the battery technology, allowing the use of T-KiBaM to model different battery types (e.g., Ni-Cd, Ni-MH or Li-ion).
- An experimental assessment with Ni-MH rechargeable batteries operating with small discharge currents for long periods of time. This experimental assessment shows that T-KiBaM achieves an average accuracy error smaller than 0.35%, when estimating the lifetime of batteries for different temperature conditions. This result significantly improves the accuracy of the original KiBaM for the same operating conditions.

This paper is organized as follows. Section 2 presents some of the most relevant related works that can be found in the literature. Section 3 addresses some fundamental concepts about battery modeling and chemical kinetics that are required for understanding the reasoning behind the proposed model. Section 4 introduces the analytical T-KiBaM and its main characteristics. Section 5 presents the performed experimental assessment that was the basis for the validation of the proposed T-KiBaM. Finally, Section 6 concludes the paper and indicates some guidelines for future work.

## 2. Related Work

Despite the widespread knowledge that thermal effects can influence the battery lifetime, analytical battery models often ignore this fact. Nevertheless, some works strive to integrate thermal effects on battery models.

Rong and Pedram [4] designed a high-level analytical model to predict the remaining capacity of a Li-ion battery. The model considers the output voltage, the discharge rate and the battery temperature and aging (number of recharge cycles). The Dualfoil simulator [23] was used as the reference for the assessment of this model. Although presenting a maximum accuracy error of 5%, the on-line prediction model requires the use of a System Management Bus (SMBus) module, which is an integrated circuit inside the battery. Besides, this model also requires multiple parameters to be tuned in (up to 42 parameters).

Szente-Varga et al. [24] proposed a set of different models for Ni-MH batteries, based on mathematical approximating functions, which consider arbitrary temperatures and discharge currents. The most appropriate model uses the sum of two hyperbolas and a linear component. It has a total of six parameters. It is possible to retrieve the values for all parameters by curve fitting, according to data retrieved from experiments. However, this model is only able to predict the behavior from a known battery discharge curve (V×t) with constant temperature and load.

Erdinc et al. [14] proposed a dynamic model for Li-ion batteries encompassing both temperature and capacity fading effects. The output voltage considers the open-circuit voltage, the voltage drop from the battery equivalent internal impedance and the temperature correction for the battery potential. The authors validated their model by comparison with other studies, assuming high battery capacities, as well as high discharge currents (>100 mA). Unfortunately, the performed experimental validation does not reflect the behavior of low-power WSN nodes.

Ye et al. [25] proposed a mathematical model for Li-ion batteries considering electronic conduction, mass transfer, energy balance and electrochemical mechanisms. In addition, the proposed model also considers the effect of temperature on battery performance. The achieved results highlight a good approximation with respect to the experimental assessment. However, the performed work was done in the context of electrical vehicles and hybrid electrical vehicles. As a result, it assesses large battery capacities (11.5 Ah) and high discharge currents (e.g., 20 A). Besides, the model has several parameters that need to be tuned in, and some of them are battery specific.

Hausmann and Depcik [26] expanded Peukert’s equation to predict the remaining battery capacity, so that it becomes possible to consider the effects of variable currents and temperatures. When comparing the obtained results with results obtained through experiments, simulation results presented a maximum error of 5% in the accuracy of remaining battery capacity prediction. Again, as the scope of the performed work was to assess the use of Li-ion batteries in vehicles, it considers high capacity batteries (60, 90 and 100 Ah) and high discharge currents (6 to 330 A).

The aforementioned research works have the following limitations when dealing with the typical usage of batteries in WSN nodes: (a) they address high battery capacities that are usually found in electrical vehicle applications; for this type of application, the focus is on the charge/discharge cycle behavior of the batteries, and not on their single-cycle charging mode and long duration operating behavior; (b) they consider that accuracy errors in the range of 5%–10% are adequate to characterize the typical usage of batteries in electrical vehicle applications; however, that is not the case for the lifetime estimation of batteries operating on single-cycle charging mode and long-term operation [7]. Other relevant works address energy issues that may be useful within the WSN context. Girban and Popa [27] discuss relevant factors that may influence the energy consumption of nodes. The authors also propose a set of analytical relationships to estimate the network lifetime. Forty et al. [28] use a node prototype to evaluate the battery lifetime in multiple experimental assessments. The achieved results indicate that the battery lifetime has a strong dependence on the time in the Rx state of the radio.

## 3. Background

This section presents some of the most relevant concepts required for the understanding of T-KiBaM analytical modeling. First, we discuss some characteristics of Ni-MH batteries, which were used to experimentally assess the proposed model. Afterwards, we present the original KiBaM, its most relevant features and operation mode. Then, we present the basics of the Arrhenius equation, used to integrate temperature dependencies in the analytical KiBaM.

### 3.1. Ni-MH Batteries

Ni-MH batteries are widely used in portable electronic devices, due to their lightweight characteristics and their high energy density. Furthermore, Ni-MH battery technology supports operation in a wide range of temperatures, usually between −10 and 45 °C.

**Figure 1:**
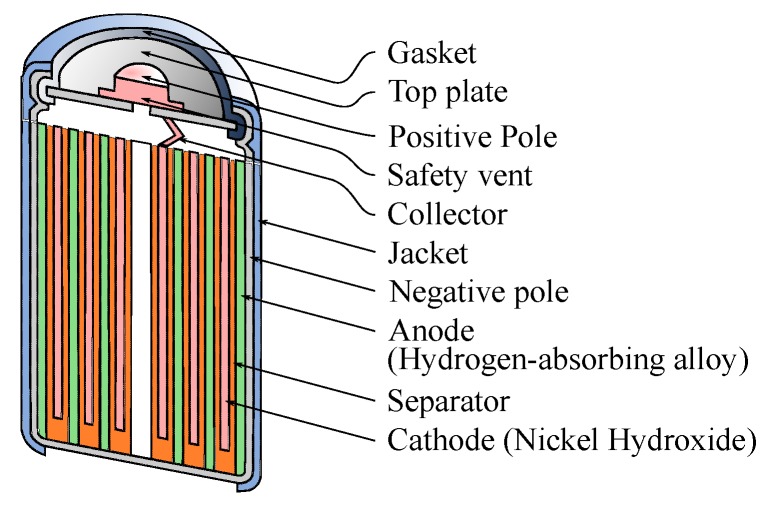
The structure of a Ni-MH battery [29].

A Ni-MH battery uses nickel hydroxide in the positive electrode. An alloy absorbs hydrogen at the negative electrode. The electrolyte composition is predominantly an aqueous solution of potassium hydroxide. Figure 1 depicts the structure of a common Ni-MH battery.

The charge and discharge chemical reactions in a regular Ni-MH battery occur as follows [29]:

- Positive electrode:

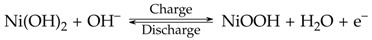
- Negative electrode:

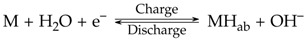

- Overall reaction:

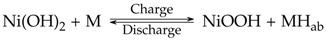

In this case, *M* is the hydrogen-absorbing alloy and $H_{ab}$ is the absorbed hydrogen. The overall reaction principle concerns the hydrogen movement from the positive to the negative electrode during the charging process, without the electrolyte taking part in the reaction. The opposite reaction occurs during the discharge process.

A Ni-MH battery presents five important characteristics [29]: (i) The charge procedure is influenced by current, time and temperature, which modifies the battery voltage behavior; the charge procedure can be sped up by higher currents or lower temperatures. Temperatures between 0 °C and 40 °C are suitable for the charging procedure, with a current of 1C (the value of 1C expresses a multiple of the battery nominal capacity) or less; (ii) The discharge procedure is influenced by the same factors. The discharge curve is flat at 1.2 V (assuming only one battery cell), presenting an efficiency reduction if the current increases or if the temperature decreases; (iii) The storage for long periods of time often causes capacity loss due to an effect known as self-discharge. This effect is influenced by the temperature at which the battery is stored. Thus, self-discharge increases with higher temperatures or long storage periods; (iv) The battery life cycle depends on several factors, such as temperature, discharge current, storage conditions, etc. Generally, a Ni-MH battery has a lifetime cycle longer than 500 recharge cycles, if used appropriately over time; (v) The safety of a battery cell must be protected against overload, short circuit and reverse charge. In the case of any of these events, the self-sealing vent must be opened to prevent battery damage.

### 3.2. KiBaM

**Figure 2:**
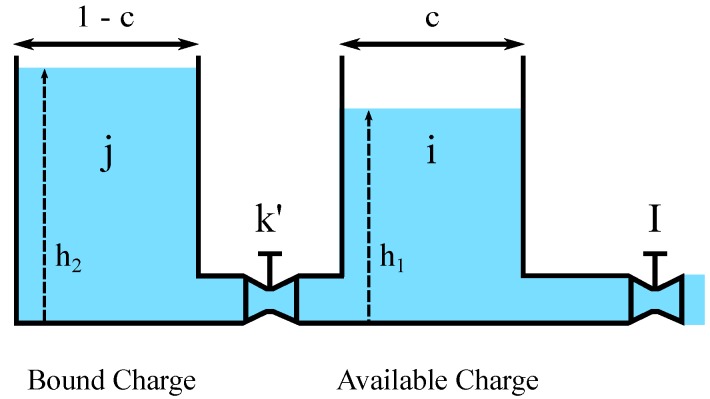
Kinetic Battery Model (KiBaM) [18].

KiBaM is an analytical battery model, that was proposed by Manwell and McGowan [18,22,30] to model the behavior of high-capacity lead-acid batteries. It uses an intuitive approach, based on a two-tank analogy, to describe the charge/discharge processes. Figure 2 illustrates the abstraction used by the KiBaM, including the related variables.

The available charge tank holds an electrical charge that can be immediately used for a device draining current *I*. The bound charge tank holds a bounded charge that can flow towards the available charge tank, regulated by a valve with a fixed conductance $k^{\prime }$. Such a constant corresponds to the rate of a chemical diffusion/reaction processes. Constant *c* corresponds to the total charge ratio stored in the available charge tank. A battery is exhausted when its available charge tank becomes empty, whatever the state of the bound charge tank. The transfer of charge, as well as the amount of unavailable charge are proportional to the difference of the heights between both tanks, $\delta =(h_{2}-h_{1})$. Thus, a smaller difference between these two height values provides a longer lifetime for the battery [31]. The following system of differential equations describes KiBaM.

(1) $$\left\{\begin{array}{c} \frac{di}{dt}=-I+k^{\prime }\cdot \left(h_{2}-h_{1}\right)\\ \frac{dj}{dt}=-k^{\prime }\cdot \left(h_{2}-h_{1}\right), \end{array}\right.$$

where *i* is the available charge and *j* the bound charge. The height values are calculated as $h_{1}=i/c$ and $h_{2}=j/\left(1-c\right)$, respectively. A new rate constant *k* for the chemical diffusion/reaction process is defined as:

(2) $$k=\frac{k^{\prime }}{c\cdot (1-c)}.$$

Substituting $h_{1}$, $h_{2}$ and $k^{\prime }$ in the system of differential Equations (1), the following is obtained:

(3) $$\left\{\begin{array}{c} \frac{di}{dt}=-I-k\cdot \left(1-c\right)\cdot i+k\cdot c\cdot j\\ \frac{dj}{dt}=+k\cdot \left(1-c\right)\cdot i-k\cdot c\cdot j. \end{array}\right.$$

Laplace transforms can be used to solve this system of differential equations [18]. Thus:

(4) $$\left\{\begin{array}{c} i=i_{0}\cdot e^{-k\cdot t}+\frac{\left(y_{0}\cdot k\cdot c-I\right)\cdot \left(1-e^{-k\cdot t}\right)}{k}-\frac{I\cdot c\cdot (k\cdot t-1+e^{-k\cdot t})}{k}\\ j=j_{0}\cdot e^{-k\cdot t}+y_{0}\cdot \left(1-c\right)\cdot \left(1-e^{-k\cdot t}\right)-\frac{I\cdot \left(1-c\right)\cdot \left(k\cdot t-1+e^{-k\cdot t}\right)}{k}, \end{array}\right.$$

where $i_{0}$ and $j_{0}$ are the amount of charge in the available charge and bound charge tanks, respectively, at the beginning of the calculations (t=0). In addition, $y_{0}=i_{0}+j_{0}$, where $y_{0}$ is the amount of charge in the battery at t=0, i.e., the initial battery capacity.

In KiBaM, the unavailable charge (*u*) is given by Equation 5, where *δ* is the difference between heights [31]. Equation (6) describes how to compute this difference [21].

(5) u=(1−c)·δ

(6) $$\delta =\left(h_{2}-h_{1}\right)=\frac{j}{\left(1-c\right)}-\frac{i}{c}.$$

The reasoning behind the use of *δ* is to capture the non-linear capacity variation of the battery [32]. Thereby, KiBaM can model two important battery effects:

- Rate capacity refers to the applied discharge current intensity, i.e., a larger discharge current implies faster battery discharges and, therefore, reduces its lifetime. This is due to the battery voltage level, which decays slowly during the battery discharge, reducing its effective capacity for higher discharge currents [7].
- Charge recovery refers to the ability of a battery to partially recover its charge during an idle period, after a discharge period. This phenomenon is related to the electrochemical stabilization inside the battery pack.

**Figure 3:**
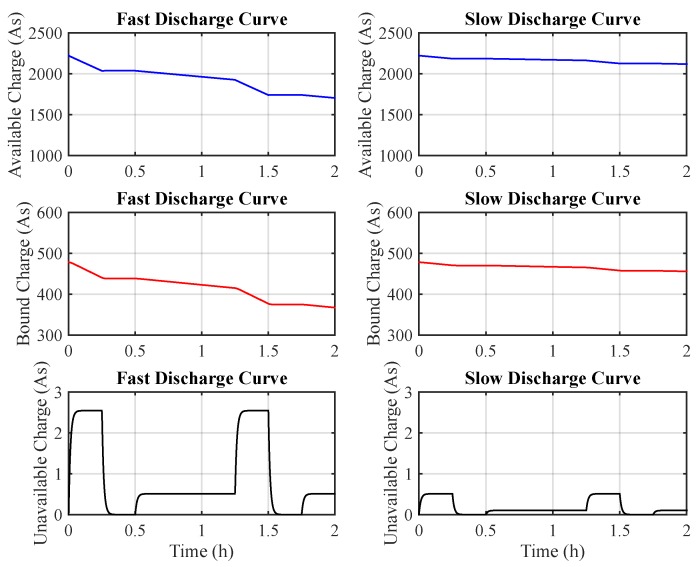
An example of KiBaM behavior over time.

The non-linear battery behavior is highly visible, particularly when these two effects act together. This is the case of batteries powering-up WSN nodes that operate in duty cycle schemes, i.e., shorter periods during which the radio is in normal operation (high discharge currents) and longer periods during which it is in low power or sleep mode (low discharge currents). Figure 3 depicts an example of a WSN battery modeled by KiBaM regarding the behavior of both tanks: available charge and bound charge. In the fast discharge curve, the following tasks are performed: 250 mA (900 s), 100 μA (900 s) and 50 mA (2700 s). In the slow discharge curve, the following tasks are performed: 50 mA (900 s), 100 μA (900 s) and 5 mA (2700 s). In this example, KiBaM parameters ($y_{0}$, *c* and *k*) were adjusted to model the behavior of a Ni-MH battery.

### 3.3. Estimating KiBaM Parameters

As mentioned in [18], one of the major advantages of the KiBaM analytical model is the ability to retrieve its main parameters through a series of experimental measurements with constant discharge currents. These parameters are: the total charge ratio stored in the available tank (*c*); the charge flow rate between both tanks (*k*); and the maximum capacity of the battery ($y_{max}$). For the sake of convenience, the guidelines for obtaining these parameters are reproduced in the following paragraphs.

First, it is necessary to normalize the capacities according to a slow discharge rate, which corresponds to a discharge time $t_{2}$. This value is required to express data in terms of the ratio between capacities as follows:

(7) $$F_{t_{1},t_{2}}=\frac{y_{t_{1}}}{y_{t_{2}}},$$

where $y_{t}$ is the discharge capacity at discharge time *t*. Considering that the battery is fully charged at the beginning of the experiments, $i=y_{0}\cdot c$ and $j=y_{0}\cdot \left(1-c\right)$, then: i/j=c/(1−c). In this condition, Equation (4) representing the available charge (*i*) is modified to:

(8) $$i=y_{max}\cdot c-\frac{I\cdot \left(1-e^{-k\cdot t}\right)\cdot \left(1-c\right)}{k}-I\cdot c\cdot t.$$

Note that $y_{max}$ is the maximum capacity of the battery. It is possible to find the discharge current responsible for emptying the battery in time *t*, $I_{t}$, in terms of *c* and *k*, by considering i=0 in Equation (8). Thus:

(9) $$I_{t}=\frac{y_{max}\cdot c\cdot k}{\left(1-e^{-k\cdot t}\right)\cdot \left(1-c\right)+k\cdot c\cdot t}.$$

The total charge supplied by the battery at a given rate is the rate multiplied by its time of operation. Therefore:

(10) $$F_{t_{1},t_{2}}=\frac{t_{1}\cdot I_{t_{1}}}{t_{2}\cdot I_{t_{2}}}.$$

By replacing Equation (9) in Equation (10), after simplifying the result, it is possible to obtain the following:

(11) $$F_{t_{1},t_{2}}=\frac{t_{1}}{t_{2}}\left[\frac{\left(1-e^{-k\cdot t_{2}}\right)\cdot \left(1-c\right)+k\cdot c\cdot t_{2}}{\left(1-e^{-k\cdot t_{1}}\right)\cdot \left(1-c\right)+k\cdot c\cdot t_{1}}\right].$$

Thus, the *c* and *k* parameters can be found given any two $F_{t_{1},t_{2}}$. By rewriting Equation (11), it is possible to obtain *c* as a function of *k* (here, $F_{t}=F_{t_{1},t_{2}}$ for easy understanding):

(12) $$c=\frac{F_{t}\cdot \left(1-e^{-k\cdot t_{1}}\right)\cdot t_{2}-\left(1-e^{-k\cdot t_{2}}\right)\cdot t_{1}}{F_{t}\cdot \left(1-e^{-k\cdot t_{1}}\right)\cdot t_{2}-\left(1-e^{-k\cdot t_{2}}\right)\cdot t_{1}-k\cdot F_{t}\cdot t_{1}\cdot t_{2}+k\cdot t_{1}\cdot t_{2}}.$$

With Equation (12), the *c* and *k* values’ definition occurs when the same value of *k*, at two different discharge rates, produces a unique value of *c*. The method of least squares can be used to solve Equation (11), whenever multiple values of $F_{t_{1},t_{2}}$ are known.

Any slow discharge rate can be used to find $y_{max}$. Thus, the maximum battery capacity may be obtained from Equation (9):

(13) $$y_{max}=\frac{y_{t}\cdot \left\{\left(1-e^{-k\cdot t}\right)\cdot \left(1-c\right)+k\cdot c\cdot t\right\}}{k\cdot c\cdot t}.$$

### 3.4. Voltage KiBaM

As stated in [18], KiBaM is also able to track the battery voltage (*V*) over time. In this case, it becomes necessary to consider the internal resistance of the battery, $R_{0}$:

(14) $$V=E-I\cdot R_{0},$$

where *E* is the internal voltage of the battery. For the battery discharge case, the following equation must be used:

(15) $$E=E_{min}+\left(E_{0,d}-E_{min}\right)\frac{i}{i_{max}},$$

where $E_{min}$ is the minimum allowed internal discharge voltage (“empty”), $E_{0,d}$ is the maximum internal discharge voltage (“full”) and $i_{max}$ is the maximum capacity of the available charge tank. The internal resistance, $R_{0}$, can be experimentally determined using constant discharge currents. Its value is represented by the slope dV/dI, when the battery is fully charged, i.e., plotting V×I and finding the slope gives $R_{0}$ [18].

### 3.5. Arrhenius Equation

As noted by Manwell and McGowan [18], most chemical processes are sped up at higher temperatures, which corresponds to a higher *k* value in KiBaM. This behavior is consistent with the higher battery capacities observed at higher temperatures, suggesting that it may be appropriate to use a chemical kinetics analysis based on the Arrhenius equation to model the influence of temperature on batteries.

Svante August Arrhenius (1859–1927) has contributed to the development of classical chemical kinetics. His contribution refers to the influence of temperature on the rate of a chemical reaction, which follows an empirical law known as the Arrhenius equation:

(16) $$k=A\cdot e^{-\frac{E_{a}}{R\cdot T}},$$

where *k* is the constant rate of a reaction, *A* is the pre-exponential factor or pre-factor (in s^−1^), $E_{a}$ is the activation energy (in KJ/mol), *R* is the universal gas constant (8.314 × 10^−3^ KJ/mol·K) and *T* is the temperature (in Kelvin).

Equation (16) indicates that the increase of the reaction rate occurs either by increasing the temperature or by decreasing the activation energy (i.e., using a catalyst). In an extreme situation, i.e., an infinite temperature or the activation energy equal to zero, $e^{-\frac{E_{a}}{R\cdot T}}=1$. The result leads to k=A, which means that the value is an *A* upper-bound for the reaction rate.

Equation (16) can be written in a more convenient form by applying a natural logarithm:

(17) $$\begin{array}{cc} ln(k) & =ln\left(A\right)-\frac{E_{a}}{R\cdot T}. \end{array}$$

Typically, the activation energy ($E_{a}$) definition refers to the minimum energy required to start a chemical reaction. Experiments at two different temperatures allow one to obtain the value of the activation energy. Equation (17) can be used for both experiments. In this case, consider the following:

$$\begin{array}{cc} ln(k_{1}) & =ln\left(A\right)-\left(E_{a}/R\cdot T_{1}\right)\\ ln(k_{2}) & =ln\left(A\right)-\left(E_{a}/R\cdot T_{2}\right). \end{array}$$

It is possible to re-write the above equations as follows:

$$\begin{array}{cc} ln\left(k_{1}\right)+\frac{E_{a}}{R\cdot T_{1}} & =ln\left(k_{2}\right)+\frac{E_{a}}{R\cdot T_{2}}\\ ln\left(k_{2}\right)-ln\left(k_{1}\right) & =\frac{E_{a}}{R}\left(\frac{1}{T_{1}}-\frac{1}{T_{2}}\right). \end{array}$$

By solving both equations with respect to $E_{a}$, it is possible to obtain that:

(18) $$\begin{array}{cc} E_{a} & =\frac{R\cdot \;ln\left(\frac{k_{2}}{k_{1}}\right)}{\frac{1}{T_{1}}-\frac{1}{T_{2}}}. \end{array}$$

Furthermore, by determining the value of *k* at different temperatures, it becomes possible to find the upper-bound value for the reaction rate (*A*) through the Arrhenius plot (Equation (16)).

## 4. T-KiBaM

The target of this section is to detail the integration of the Arrhenius equation with KiBaM. The resulting analytical model, Temperature-Dependent KiBaM (T-KiBaM), is able to integrate the effect of temperature on the battery operating behavior, particularly upon both its voltage behavior and its lifetime. Consider the proposition below.

**Proposition** **1.** *Both k parameters from KiBaM and the Arrhenius equations refer to a constant reaction rate, which models the rate of a chemical diffusion/reaction process (in KiBaM, this rate is represented by the charge rate between both tanks). Thus, considering that $k_{\mathrm{KiBaM}}=k_{\mathrm{Arrhenius}}$:*

(19) $$k_{\mathrm{KiBaM}}=A\cdot e^{-\frac{E_{a}}{R\cdot T}}.$$

Therefore, the constant rate parameters of KiBaM (Equation (2)) are now re-defined to consider the activation energy ($E_{a}$) and the temperature (*T*), as defined in Equation (16).

A set of experimental assessments was performed to validate this procedure and to illustrate how to use the proposed T-KiBaM. Briefly, a set of experiments with Ni-MH batteries were performed to obtain the T-KiBaM parameters, *c* and *k*. Then, the described methodology was applied to obtain the Arrhenius constants, $E_{a}$ and *A*. Finally, the proposed approach was validated by comparing the experimental results against the results obtained with the analytical T-KiBaM. This methodology is presented below. Note that, except when explicitly stated, all presented graphs contain interpolated voltage curves upon the experimental data, in order to clearly present the obtained results.

### 4.1. Finding Activation Energy ($E_{a}$) and the Upper-Bound of the Reaction Rate (A)

The experimental assessment was done using a set of twelve Panasonic batteries, Model HHR-4MRT/2BB (2xAAA, Ni-MH, 2.4 V, 750 mAh). The batteries were fully charged at the beginning of the experiments, being discharged until reaching the cut-off value of 2.0 V. The time required for reaching this voltage level defines the battery lifetime in each experimental assessment. Note that this cut-off value is commonly used in experimental tests with Ni-MH batteries, in order to prevent the internal damage that could occur if the voltage level went below 2.0 V [29]. The recharging time was around eight hours, by using a common Ni-MH battery charger (output: 1.2 V_DC_, 250 mA). The batteries stayed at rest during, at least, 60 min before the start of each experiment.

**Figure 4:**
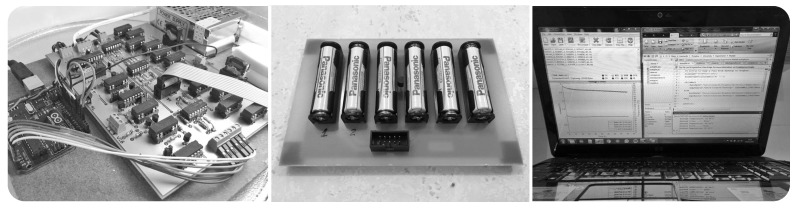
Test-bed used for the experimental assessments.

**Figure 5:**
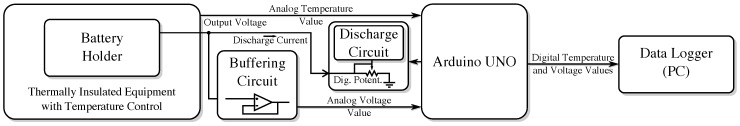
Connectivity scheme of the test-bed system.

A test-bed has been specifically developed for this experimental assessment, which includes a discharge-controlled circuit and an Arduino UNO [33]. This test-bed allows setting up controlled discharge currents to the batteries and collecting the experimental data for further analysis. Figure 4 presents a photo of the test-bed circuit, and Figure 5 illustrates the interconnection of its main blocks.

The discharge-controlled circuit is able to consume currents in the range of 30 μA–30 mA, commonly found in COTS low-power WSN nodes, e.g., MICAz from Crossbow [34]. It is possible to select among 256 consumed current values, by using a digital potentiometer (AD5206, Analog Devices, Inc., Norwood, MA, USA) [35]. The discharge-controlled circuit guarantees a constant discharge current from the batteries, by using a controlled current source. The batteries’ board also includes a temperature sensor (Maxim 18B20) [36] for temperature measurements.

An Arduino UNO controls all circuit components and collects the experimental data from the batteries’ board: battery voltage and temperature. The UNO board has analog inputs with 10-bit resolution, the data log interval being adjustable. For the performed assessments, data were recorded every 10 s. A computer receives the collected data from the UNO board through a USB connection using CoolTerm [37], which stores all data in a TXT file for further analysis.

The experiments were performed at a set of different temperatures, with a 15 °C step, −5, 10, 25 and 40 °C, using thermally-insulated equipment with temperature control. A complementary full experiment was performed for 32.5 °C, as this temperature was found to be the most relevant outlier between the assessed temperature values. For all of the experiments, only the batteries (including the temperature sensor) remained inside the thermally-insulated equipment, at a controlled temperature.

Three experimental assessments were performed for each of the above-mentioned temperature values and for each of the following discharge current values: 10, 20 and 30 mA (actually, due to the available 256 resistance values obtained from the digital potentiometer, the considered current values were: 10.424, 20.303 and 30.242 mA). A total of 45 experiments were performed, three for each current/temperature pair. Thus, average lifetime/voltage values from three experiments were considered for each measurement presented in this paper.

**Figure 6:**
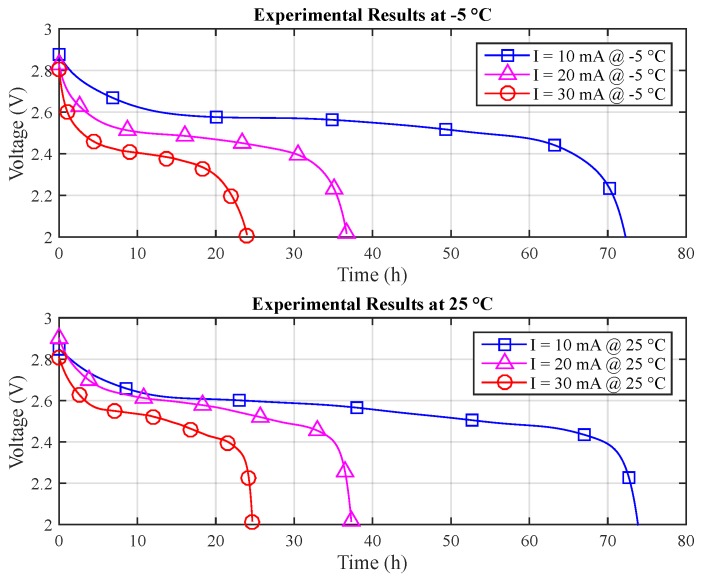
Experimental results at different temperatures.

For the estimation of the T-KiBaM parameters, only two temperature values are required. Therefore, just the measurements for −5 and 25 °C were considered for this purpose. It is possible to obtain the capacity provided by the battery using ∫Idt. As the discharge current is constant, the capacity is obtained by just multiplying the current value by the experimental lifetime, i.e., $I\cdot t_{I}$. Table 1 shows the obtained results for both temperature ranges. Figure 6 depicts the discharge curves for each temperature.

The obtained set of experimental measurements (numerical values are represented with up to five significant figures, whenever available) allows the evaluation of the T-KiBaM parameters, as previously explained in Section 3.3:

$$\begin{array}{c} T_{1}=268.15\;K:\;c_{1}=0.56350,\;k_{1}=0.56401;\\ T_{2}=298.15\;K:\;c_{2}=0.56486,\;k_{2}=0.59526. \end{array}$$

Note that being that $k_{1}<k_{2}$, this indicates that the reaction rate is slower at lower temperatures. By using Equation (18), it is possible to obtain the activation energy value, $E_{a}$, which is equal to 1.1949KJ/mol. Through the activation energy ($E_{a}$), $k_{2}$ and temperature ($T_{2}$) values, it is possible to obtain an upper-bound for the reaction rate (*A*):

(20) $$\begin{array}{cc} k_{2}=\; & A\cdot e^{-\frac{E_{a}}{R\cdot T_{2}}}\\ A=\; & 0.96397\;s^{-1}. \end{array}$$

The new values for *k* are then obtained by combining Equation (19) with the obtained values of *A*, $E_{a}$, *R* and *T*. Thus, *k* values vary with temperature, defining the T-KiBaM dependence on temperature as shown by Proposition 1. Finally, *A* and $E_{a}$ values are constant values for a given battery type and do not depend on the temperature value. Table 2 depicts the relationship between *k* and temperature.

Note that it is possible to extrapolate the values of *k* beyond the temperature range at which the parameters were obtained, due to the chemical kinetics concepts modeled by the Arrhenius equation. Table 2 shows the behavior of the reaction rate (*k*) between −12.5 and 47.5 °C. However, it is also worth mentioning that the operating limits of the battery must be taken into account for these extrapolations. The indicated temperature range for the discharge of Ni-MH batteries varies from −10 to 45 °C [29].

### 4.2. Calibrating T-KiBaM for Battery-Specific Characteristics

Temperature also affects the battery capacity. Typically, batteries provide higher effective capacities at higher temperatures [2] and lower effective capacities when used at lower temperatures [38]. Within this context, it is crucial to adjust T-KiBaM to the technology of the battery being modeled (e.g., Ni-MH or Li-ion).

Therefore, in this section, we present a method for adjusting the T-KiBaM parameters accordingly. Briefly, through a set of experimental assessments, it is possible to evaluate the losses and gains of the battery capacity according to the temperature variation. Such knowledge is incorporated in T-KiBaM to model the initial battery capacity under different temperature conditions.

**Figure 7:**
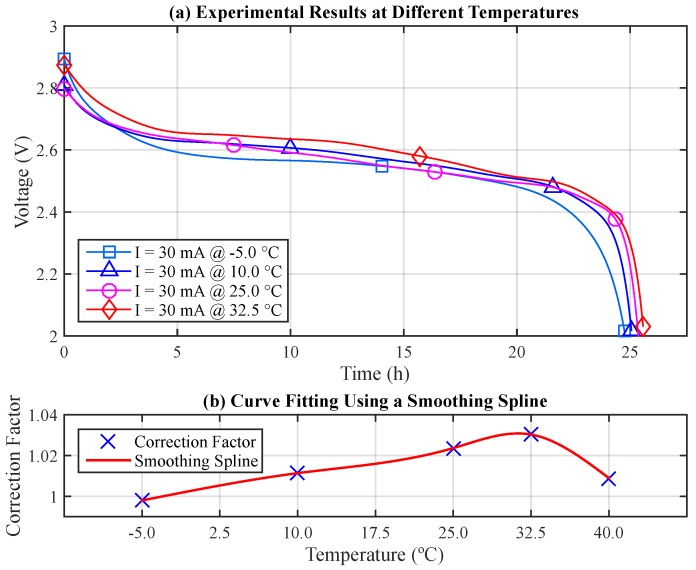
The battery-specific behavior. (**a**) Experimental results; (**b**) Curve fitting.

First, fifteen experiments were performed with the same discharge current, 30 mA, under five different temperatures: −5, 10, 25, 32.5 and 40 °C (three experiments for each temperature). Each of the following average lifetimes was obtained from three battery pairs: 24.749, 25.087, 25.385, 25.560 and 25.022 h, respectively. Figure 7a illustrates the related discharge curves, V×t (for the sake of clarity, the results at 40 °C were not included). Note the non-linearity of these voltage curves throughout the experiments with different curves for different temperatures, even using the same discharge current.

As previously mentioned, the capacity provided by the battery can be evaluated by integrating its discharge current over time. From the obtained results, it becomes possible to evaluate the losses and gains of the battery capacity at different temperatures, in order to establish the Correction Factor (CF) of the initial battery capacity ($y_{0}$) for each situation. The losses and gains of the battery capacity are evaluated with respect to the battery nominal capacity, which was 750 mAh for this case. Table 3 presents the obtained results.

After establishing the correction factor, which indicates the gain or loss of the initial battery capacity ($y_{0}$) at different temperatures, it is possible to find a function that properly fits the data. Figure 7 (b) depicts the CF data points and the fitted curve. A smoothing spline (piecewise polynomial function of degree three) [39] with p=0.6 and w=[1,1,1,1,1] fits the obtained data points:

$$\mathrm{CF}\left(T\right)=a\cdot {\left(T-T_{1}\right)}^{3}+b\cdot {\left(T-T_{1}\right)}^{2}+c\cdot {\left(T-T_{1}\right)}^{1}+d,$$

where $T_{1}\leq T<T_{2}$. Table 4 presents the coefficients for each segment. Note that this function enables the adjustment of the initial battery capacity according to the selected temperature and is valid only within the aforementioned temperature range, i.e., from −5 to 40 °C, which represents the case for most part of the applications within the WSN context (e.g., from snow [40] to industrial [41] monitoring applications).

### 4.3. Modeling the Voltage Behavior for Ni-MH Batteries

Voltage is important output information from the battery modeling, as it provides a perspective on how the battery behaves over time. In this sense, it is valuable to model variable V×t during the discharge process, in order to understand the ability of the battery to maintain its nominal voltage. The original KiBaM offers this possibility (Equation (14)), though inaccurately since it does not model the exponential voltage decrease at the beginning of the discharge curve, nor the “knee” at the end of the discharge curve (as will be illustrated later, in this section), which can be observed in real experiments with Ni-MH batteries (cf. Figure 7). Thus, it becomes necessary to improve the voltage model that has been used in KiBaM.

Tremblay and Dessaint [42,43] developed a voltage model for different battery technologies, e.g., lead-acid, Ni-MH, Ni-Cd and Li-ion. Although being able to handle both charge and discharge curves for each battery type, only the Ni-MH battery discharge model is presented in this paper.

The Tremblay–Dessaint voltage model can accurately represent the voltage dynamics with varying current values. Besides, it considers the Open Circuit Voltage (OCV) as a function of the State of Charge (SoC). Thus, the battery voltage may be obtained as follows:

(21) $$V_{b}=E_{0}-K_{b}\cdot \frac{Q}{Q-it}\cdot it-R_{b}\cdot i+A_{b}\cdot e^{\left(-B\cdot it\right)}-K_{b}\cdot \frac{Q}{Q-it}\cdot i^{*},$$

where $V_{b}$ is the battery voltage (V), $E_{0}$ is the battery constant reference voltage (V), $K_{b}$ is the polarization resistance (Ω), *Q* is the battery capacity (Ah), it=∫idt is the actual battery charge (Ah), $A_{b}$ is the exponential zone amplitude (V), *B* is the exponential zone time constant inverse (Ah)^−1^, $R_{b}$ is the internal resistance (Ω), *i* is the discharge current (A) and $i^{*}$ is the filtered current (A). For further details, please refer to [42,43].

Equation (21) is valid only for Li-ion batteries, as it presents an exponential term that is not observed in other battery types, such as lead-acid, Ni-MH and Ni-Cd. These batteries exhibit a hysteresis phenomenon between the charge and discharge processes, which occurs only at the beginning of the discharge curve, regardless of their SoC. This phenomenon can be represented by a non-linear dynamic system:

(22) $$\dot{\mathrm{Exp}}\left(t\right)=B\cdot \left|i\left(t\right)\right|\cdot \left(-\mathrm{Exp}\left(t\right)+A_{b}\cdot u\left(t\right)\right),$$

where Exp(t) is the exponential zone voltage (V), i(t) is the discharge current (A) and u(t) is the charge/discharge mode. The exponential voltage relies on its initial value $\mathrm{Exp}(t_{0})$ and the charge (u(t)=1) or discharge (u(t)=0) mode.

Briefly, the final form for the discharge equation of the voltage model for Ni-MH and Ni-Cd batteries is as follows:

(23) $$V_{b}=E_{0}-R_{b}\cdot i-K_{b}\cdot \frac{Q}{Q-it}\cdot \left(it+i^{*}\right)+\mathrm{Exp}\left(t\right).$$

However, this voltage model presents an inconsistency at the end of the analytical evaluation (cf. Figure 4 of [42]), where the following problems arise: (P1) the analytical lifetimes are smaller than the experimental lifetimes at the battery voltage cut-off point; and (P2) the model returns voltage values below zero for time instants close to the end of the analytical evaluation.

#### Temperature-Dependent Voltage Model (TVM)

In this section, we propose the Temperature-Dependent Voltage Model (TVM) model, an extension of the Tremblay–Dessaint model, which is able to attenuate the above described problems (P1 and P2) and appropriately represent the influence of the temperature on the V×t curve during the battery discharge.

In order to increase the accuracy of the Tremblay–Dessaint voltage model, i.e., attenuating P1 and P2, a smoothing constant, $\tau_{b}$, has been added. This $\tau_{b}$ value multiplies the terms that relate current and time, i.e., it and $\dot{\mathrm{Exp}}\left(t\right)$. Therefore, Equations (22) and (23) should be rewritten as follows:

(24) $$\dot{\mathrm{Exp}}\left(t\right)=\tau_{b}\cdot B\cdot \left|i\left(t\right)\right|\cdot \left(-\mathrm{Exp}\left(t\right)+A_{b}\cdot u\left(t\right)\right),$$

(25) $$V_{b}=E_{0}-R_{b}\cdot i-K_{b}\cdot \frac{Q}{Q-it\cdot \tau_{b}}\cdot \left(it\cdot \tau_{b}+i^{*}\right)+\mathrm{Exp}\left(t\right).$$

All parameters ($A_{b}$, *B*, $E_{0}$, *Exp*$\left(t_{0}\right)$, $K_{b}$, *Q*, $R_{b}$ and $\tau_{b}$) can be obtained using the battery data-sheet or by performing experimental measurements [43]. Considering an experiment at −5 °C, for example, the following values can be obtained:
$A_{b}=0.2831\;$V
$\tau_{b}=0.954$
$E_{0}=2.570\;$VB=18(Ah)${}^{-1}$$K_{b}=0.0375\;\Omega$$\mathrm{Exp}(t_{0})=0.280\;$V$R_{b}=0.070\;\Omega$Q=0.75Ah·*CF*(−5).

**Figure 8:**
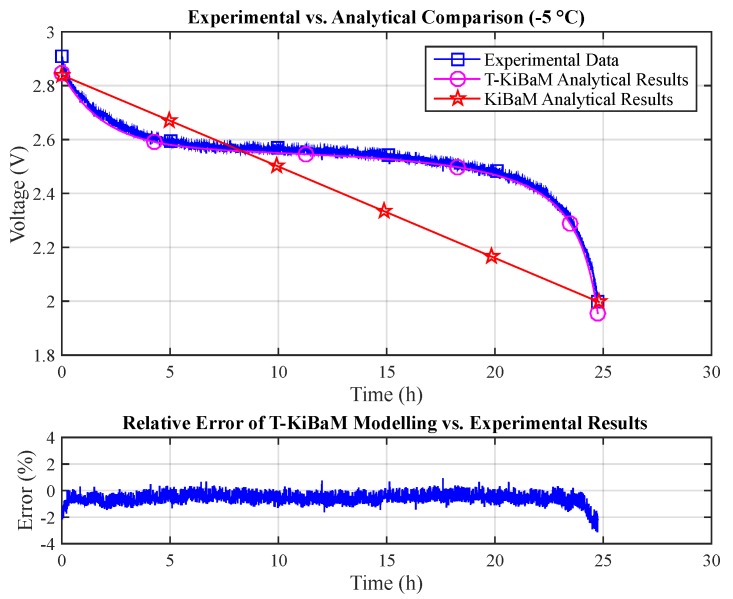
Experimental vs. analytical results.

Figure 8 compares data obtained through the experimental assessment (*T* = −5 °C, *I* = 30 mA) with both the T-KiBaM (Equation (25)) and the original KiBaM analytical results (Equation (14)). The raw experimental data are then used to make a point-to-point comparison with just the T-KiBaM analytical curve, as the original KiBaM linear results are clearly inaccurate along the time scale. Note that a reduced steady-state relative error is obtained for most of the T-KiBaM analytical results. The following absolute errors were obtained for T-KiBaM values: 3.1% (max) and 0.6% (mean).

Experiments performed for different temperature values enabled the extraction of the voltage model parameters. Table 5 illustrates the Arrhenius constants obtained for each parameter, as well as the values of all parameters at each temperature.

**Figure 9:**
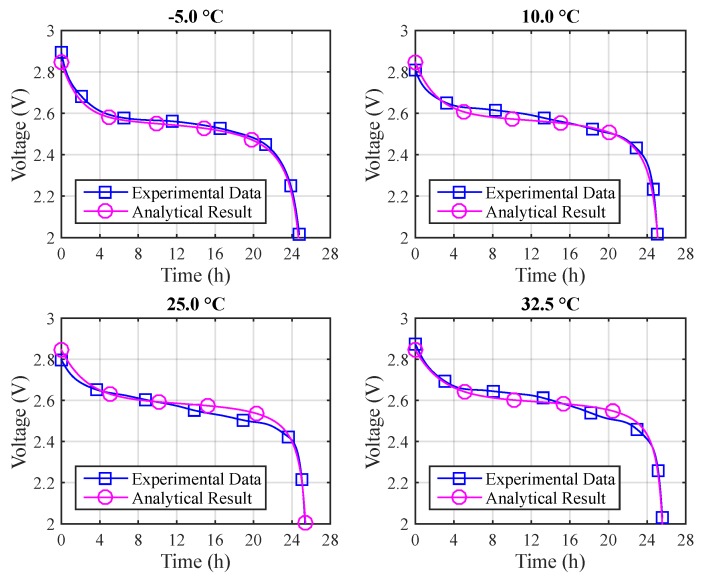
Experimental vs. T-KiBaM analytical results at different temperatures.

**Figure 10:**
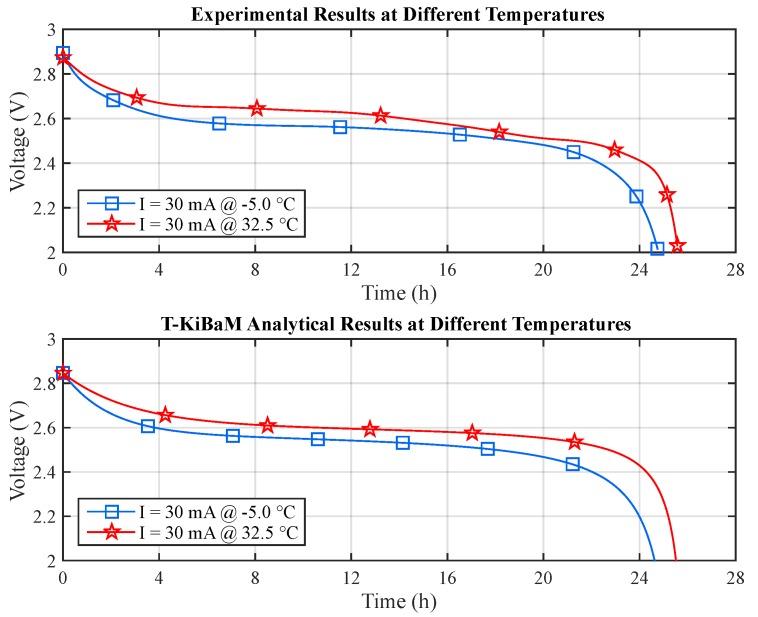
Experimental vs. T-KiBaM analytical comparison at different temperatures.

Finally, it becomes possible to represent the values obtained from the T-KiBaM analytical voltage model adapted to different temperatures, using the set of parameters represented in Table 5, along with their respective constants *A* and $E_{a}$, using the Arrhenius equation. Figure 9 depicts the voltage results for the different temperatures using Arrhenius constants. The absolute errors are: (a) *T* = −5 °C: 3.1% (max) and 0.6% (mean); (b) *T* = 10 °C: 2.9% (max) and 0.7% (mean); (c) *T* = 25 °C: 2.0% (max) and 0.9% (mean); (d) *T* = 32.5 °C: 6.7% (max) and 1.2% (mean). Figure 10 depicts a comparison between the experimental and the T-KiBaM analytical results, regarding the voltage levels at different temperatures.

The modified voltage model, which is called TVM, was integrated into T-KiBaM, as it satisfactorily represents the battery discharge behavior regarding the voltage level, for the case of Ni-MH batteries at different temperatures.

### 4.4. T-KiBaM Summary

This section summarizes the parameters of the T-KiBaM analytical model regarding the used Ni-MH batteries. Table 6 illustrates the experimentally-obtained values, as described in the previous sections (in Table 6; $T_{c}$ and $T_{k}$ are the temperatures in degrees Celsius and Kelvin, respectively).

## 5. Analytical and Experimental Results

Finally, this section aims to present the performed T-KiBaM validation. Briefly, we use MATLAB to implement the T-KiBaM analytical model (Equations (4) and (19)). Then, both analytical and experimental results were compared regarding the battery lifetime. Below, details about the implementation of the analytical set-up are shown.

### 5.1. Implementing T-KiBaM

First, the following steps must be performed to find the function that allows calculating the Correction Factor (CF) with respect to the initial capacity of the battery, according to the temperature (TEMP):

1. Perform at least four experiments at distinct temperatures using the same constant discharge current, whose value should be within the interest range of the supported WSN application.
2. From the obtained results in Step 1, extract the charge losses/gains according to the battery nominal capacity.
3. From TEMP vs. CF data, it is possible to determine a function that properly fits the data behavior.
4. Add such a function to the T-KiBaM implementation.

Next, there is the need to implement Equation (4) as a function to compute the battery lifetime. Its purpose is to update the content of both tanks: available charge and bound charge. Algorithm 1 depicts the function structure, as well as its definitions.
 **Algorithm 1:** T-KiBaM_function  **Input**: *c*, *k*, $i_{0}$, $j_{0}$, $t_{0}$, *I*, $t_{I}$  **Output**: $i_{0}$, $j_{0}$, $t_{0}$**1**$y_{0}=i_{0}+j_{0}$;**2**$t_{0}=t_{0}+t_{I}$;**3**$i_{0}$ = compute-i ($c,k,y_{0},i_{0},j_{0},I,t_{I}$);**4**$j_{0}$ = compute-j ($c,k,y_{0},i_{0},j_{0},I,t_{I}$);**5****return** ($i_{0},j_{0},t_{0}$);

The T-KiBaM function input parameters are: *c*, *k*, $i_{0}$, $j_{0}$, $t_{0}$ (total lifetime), *I* (discharge current) and $t_{I}$ (operating time of *I*). Note that Lines 3 and 4 perform the calculations corresponding to Equation (4). In addition, by making the appropriate changes, one-dimensional arrays can replace *I* and *t* variables to evaluate a set of tasks. Algorithm 2 depicts the call for the previously-described function.
 **Algorithm 2:** T-KiBaM_call**  ****Input**: $E_{a},A,R,T,c,y_{0},k,t_{0},I,t_{I}$**Output**: $i_{0},j_{0},t_{0}$ (Computed Lifetime)**1**$y_{0}=y_{0}\cdot \mathrm{CF}\left(T\right)$;**2**$i_{0}=\left(c\right)\cdot y_{0}$;**3**$j_{0}=\left(1-c\right)\cdot y_{0}$;**4**$k=A\cdot e^{-Ea/(R\cdot T)}$;**5****while**
*$i_{0}$ > 0*
**do****6** | [
$i_{0},j_{0},t_{0}$] = T-KiBaM_function (
$c,k,i_{0},j_{0},t_{0},I,t_{I}$);**7****end****8****return** ($i_{0},j_{0},t_{0}$);

In this case, note that the correction factor is applied in Line 1, as described in Section 4.2. In addition, the definition of *k* (Line 4) considers the Arrhenius equation values ($E_{a}$, *A*, *R* and *T*), obtained through experiments in Section 4.1. Through the while loop (Line 5), it is possible to verify the content of the available charge tank, which needs to be greater than zero, even if there is charge in the bound charge tank. Thus, regardless of the available charge in the available charge tank, the algorithm performs the discharge according to the load value (*I*).

### 5.2. Validating T-KiBaM

The T-KiBaM analytical evaluation involves the use of Algorithms 1 and 2. The following discharge currents were used for the evaluations: 20 and 30 mA, which represent the same current values used for the experimental assessments. The input parameters were the same as those mentioned in Algorithm 2. However, note that the battery initial capacity ($y_{0}$) is defined according to the values of each experiment. Besides, the value c=0.56418 was used, which represents the average of $c_{1}$ and $c_{2}$ values (mentioned in Section 4.1).

Table 7 illustrates the results of the analytical evaluations using T-KiBaM. The EXP, T-KiBaM and ERR columns represent, respectively, the experimental average lifetime of three battery measurements, the lifetime using T-KiBaM and the relative error between EXP and T-KiBaM. Note that the adapted model performs the battery lifetime estimation with an average relative error of 0.21% for low temperatures (−5 °C) and 0.23% for 25 °C.

These results demonstrate that T-KiBaM is able to accurately estimate the battery lifetime of WSN nodes, presenting average accuracy errors smaller than 0.25% when using the battery nominal capacity. Our rationale is that, if all 45 tests (with one pair of batteries each) were started with exactly the same battery capacity, even better results would have been achieved using T-KiBaM. However, this is an assumption that is difficult to hold as, due to electrochemical reactions inside the batteries, it is not possible to guarantee the exact same value with respect to the initial battery capacity for each test.

### 5.3. Model Comparison: KiBaM vs. T-KiBaM

This section compares the original KiBaM and T-KiBaM as concerns the expected battery lifetimes for five different temperatures. The target is to perform an experimental validation of T-KiBaM. The analytical evaluations consider the following details.

The constants used for setting up KiBaM are the ones that were obtained at 25 °C, i.e., $c_{2}=0.56486$ and $k_{2}=0.59526$. For T-KiBaM, c=0.56418, and the value of *k* varies according to the evaluated temperatures (cf. Table 2). The initial battery capacity is the same for both models, $y_{0}=2700$ As (750 mAh). Nevertheless, T-KiBaM adjusts this value after the start of the analytical evaluation, in accordance with the related Correction Factor (CF). The evaluated temperatures (TEMP) were the following: −5, 10, 25, 32.5 and 40 °C. The battery is drained until the end of the available charge in all of the analytical evaluations. Table 8 presents the results obtained using both models.

As expected, KiBaM presents the same battery lifetimes for all situations, regardless of the evaluated temperature. In the KiBaM analytical evaluation, the average relative error is 1.50%, with a standard deviation of 1.12%. The largest error occurs at 32.5 °C, where a difference of 2.98% (45 min) is achieved between the experimental assessment and the analytical evaluation. Considering a period of 365 days (one year), this error would generate a difference in the battery lifetime of 10.8 days. On the other hand, T-KiBaM presents different battery lifetimes according to the assessed temperatures. For this case, the average relative error is 0.01%, with a standard deviation of 0.01%. The largest error occurs also at 32.5 °C, where a difference of 0.03% (29 s) is observed. In one year, this error would represent a difference of 0.1095 days [1,44].

It is possible that thermal effects will have an even greater impact when applying intermittent discharge currents to the battery, e.g., WSN nodes operating in a duty cycle scheme. Our rationale is that, by taking advantage of the radio sleep periods at different temperatures, the transfer rate from the bound charge tank to the available charge tank (recovery effect) will also be variable, i.e., parameter *k* will have different values according to the temperature (cf. Table 2). In other words, a smaller value of *k* implies a slower rate for the charge recovery. This behavior may have a substantial impact on the battery lifetime for different temperatures, particularly when long periods of time are evaluated, e.g., weeks or months. Therefore, this is an issue that must be addressed in future work.

## 6. Conclusions

Estimating battery lifetimes is a complex task, as there are multiple factors influencing their behavior. The most studied factors are both the rate capacity and the charge recovery. However, the thermal effect also plays an important role as concerns the evaluation of their lifetime. Thermal effects can modify the electrochemical reaction rate and/or impair the battery operation. In the case of WSNs, battery-powered nodes are especially prone to the influences of temperature variations, especially in outdoor environments.

Within this context, the availability of adequate battery models able to encompass thermal effects would be of utmost importance. This paper proposes an extension to the widely-used analytical KiBaM to encompass thermal effects. As a consequence, it provides a valuable tool for the battery lifetime estimation at different temperatures. The proposed model extension was validated through an extensive experimental assessment, using Ni-MH batteries operating at different temperatures. The achieved results show that the proposed T-KiBaM extension presents an average accuracy error smaller than 0.33%, when estimating the lifetime of batteries for different temperature conditions. This result significantly improves the accuracy of KiBaM for the same operating conditions, which is slightly smaller than 2.7%. The full experimental and analytical assessments can be examined in Appendix A.

As future work, it would be necessary to evaluate the behavior of T-KiBaM with other battery types, e.g., Li-ion. The rationale beyond the proposed set of equations suggests that the same set of formulae can be used to obtain the parameters for different battery types. Furthermore, the proposed extension of KiBaM should also be tested for duty cycle battery loads, similar to those that can be found in typical WSN applications. Finally, it would be interesting to evaluate the performance of the proposed model in a microcontroller typically used in WSNs.

## Figures and Tables

**Table 1:** Experimental results.

Discharge Current (mA)	−5 °C		25 °C	
	Lifetime (h)	Capacity (mAh)	Lifetime (h)	Capacity (mAh)
10	72.31	753	73.88	770
20	36.54	741	37.36	758
30	23.99	725	24.63	744

**Table 2:** Variation of *k* according to temperature.

Temperature (°C)	*k* Value (s^−1^)	Temperature (°C)	*k* Value (s^−1^)	Temperature (°C)	*k* Value (s^−1^)
−12.5	0.55538	10.0	0.58025	32.5	0.60234
−5.0	0.56401	17.5	0.58790	40.0	0.60917
2.5	0.57229	25.0	0.59526	47.5	0.61574

**Table 3:** Correction Factor (CF) at different temperatures.

TEMP (°C)	Time (h)	Capacity (mAh)	Loss or Gain (%)	Correction Factor
−5.0	24.749	748.5	−0.2	0.9980
10.0	25.087	758.6	1.14	1.0114
25.0	25.385	767.7	2.36	1.0236
32.5	25.560	772.9	3.05	1.0305
40.0	25.022	756.7	0.89	1.0089

Discharge current equal to 30 mA.

**Table 4:** Smoothing spline coefficients.

a	b	c	d	Segment
−5.1170 × 10${}^{-7}$	0	1.0076 × 10${}^{-3}$	9.9800 × 10${}^{-1}$	−5.0 ≤ T < 10.0
2.2375 × 10${}^{-6}$	−2.3027 × 10${}^{-5}$	6.6220 × 10${}^{-4}$	1.0114	10.0 ≤ T < 25.0
−2.0925 × 10${}^{-5}$	7.7663 × 10${}^{-5}$	1.4817 × 10${}^{-3}$	1.0237	25.0 ≤ T < 32.5
1.7473 × 10${}^{-5}$	−3.9315 × 10${}^{-4}$	−8.8444 × 10${}^{-4}$	1.0303	32.5 ≤ T < 40.0

**Table 5:** Temperature-Dependent Voltage Model (TVM) parameters at different temperatures.

TVM Parameter	Temperature (°C)					Arrhenius Constants	
	−5	10	25	32.5	40	*A*	$E_{a}$
$E_{0}$	2.5700	2.5850	2.6000	2.6060	2.6120	2.884200	0.25714
$R_{b}$	0.0700	0.0480	0.0350	0.0300	0.0260	0.000071	−15.358
$K_{b}$	0.0375	0.0286	0.0225	0.0201	0.0180	0.000234	−11.318
*B*	18.000	15.010	12.750	11.820	11.000	0.584660	−7.6403
$\mathrm{Exp}(t_{0})$	0.2800	0.2620	0.2470	0.2410	0.2350	0.082728	−2.7181
$\tau_{b}$	0.9540	0.9630	0.9706	0.9742	0.9776	1.126800	0.36978

**Table 6:** Temperature-Dependent KiBaM (T-KiBaM) parameters for a Ni-MH battery.

Model	Parameter	Value
	$E_{a}$	1.1949
Arrhenius	*A*	0.96397
	*R*	0.008314
CF ($T_{c}$)	*a*, *b*, *c*, *d*	cf. Table 4
T-KiBaM	*c*	0.56418
	*k*	$A\cdot e^{\frac{-E_{a}}{R\cdot T_{k}}}$
	$y_{0}$	750 ·*CF* ($T_{c}$)
TVM	$A_{b}$, *B*, $E_{0}$, *Exp*$\left(t_{0}\right)$, $K_{b}$, *Q*, $R_{b}$, $\tau_{b}$	cf. Table 5

**Table 7:** T-KiBaM analytical results. EXP: Experimental result; ERR: Relative error.

	−5 °C			25 °C		
Discharge Current (mA)	EXP (h)	T-KiBaM (h)	ERR (%)	EXP (h)	T-KiBaM (h)	ERR (%)
20	36.714	36.866	0.41	37.984	37.815	0.44
30	24.749	24.750	0.00	25.385	25.386	0.01
Average			0.21			0.23

**Table 8:** Comparison between models.

TEMP (°C)	EXP (h)	KiBaM (h)	ERR (%)	T-KiBaM (h)	ERR (%)
−5.0	24.749	24.799	0.20	24.750	0.00
10.0	25.087	24.799	1.15	25.082	0.02
25.0	25.385	24.799	2.31	25.386	0.01
32.5	25.560	24.799	2.98	25.552	0.03
40.0	25.022	24.799	0.89	25.022	0.00
Average			1.50		0.01

Discharge current equal to 30 mA.

**Figure A1:**
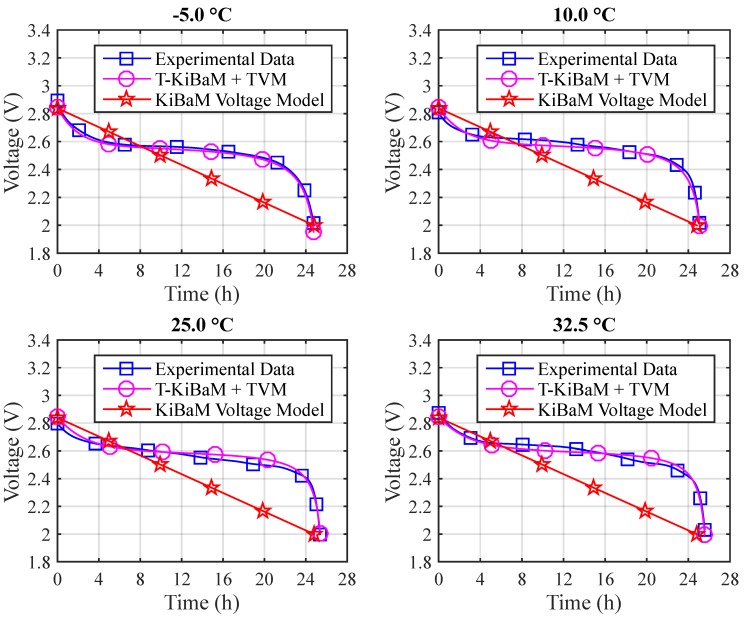
Voltage level tracking comparison.

## Appendix A. Full Analytical Results

Table A1 illustrates a comparison between experimental and analytical results for each of the assessed temperatures. The EXP column represents the average discharge time of three discharge experiments using different batteries of the same model, i.e., Panasonic HHR-4MRT/2BB.

The results obtained when using using Peukert’s Law [7,45,46,47] were also included for comparative purposes. This is a simpler analytical battery model, which can capture part of the non-linear properties of the batteries, where the battery lifetime (*L*) can be evaluated using:

(A1) $$L=\frac{a}{I^{b}},\;$$

*I* being the discharge current and *a* and *b* experimentally-obtained constants. Ideally, *a* corresponds to the battery capacity, and *b* is equal to one. In this comparison, the value of *a* was set at 0.75 Ah, which corresponds to the nominal battery capacity, and *b* was adjusted according to the experimental results at 25 °C using the discharge current *I* = 30 mA. Thus, *b* = 1.0067.

Table A1 also contains the results of T-KiBaM when the discharge current of 20 mA was used to calculate the function that returns the correction factor for each temperature. In this case, the same methodology mentioned in Section 4.2 was followed to generate the function parameters.

The behavior of the battery voltage over time was also assessed in this work. In this case, the experimental results were compared with the analytical results using both KiBaM and T-KiBaM. Figure A1 depicts some examples of voltage tracking at different temperatures.

Note that KiBaM presents the same results in all analytical assessments, since this model is not able to handle different temperatures. In addition, KiBaM represents linearly the voltage behavior, which causes a significant error in relation to the experimental data when analyzing different cut-off points, as in the range between 2.0 and 2.5 V. On the other hand, T-KiBaM is able to deal more accurately with voltage tracking at different temperatures. For instance, at *T* = −5 °C (Figure A1 top-left), analyzing the voltage level equal to 2.4 V, the relative error to the experiment of KiBaM is 37.53%, while in T-KiBaM is 0.73%.

**Table A1:** Comparison between experimental and analytical results.

	Temperature (°C)														
	−5			10			25			32.5			40		
**Discharge Current (mA)**	**EXP (h)**	**Peukert’s Law (h)**	**ERR (%)**	**EXP (h)**	**Peukert’s Law (h)**	**ERR (%)**	**EXP (h)**	**Peukert’s Law (h)**	**ERR (%)**	**EXP (h)**	**Peukert’s Law (h)**	**ERR (%)**	**EXP (h)**	**Peukert’s Law (h)**	**ERR (%)**
20	36.714	37.91	3.27	37.402	37.91	1.37	37.984	37.91	0.19	37.835	37.91	0.21	37.133	37.91	2.10
30	24.749	25.39	2.57	25.087	25.39	1.19	25.385	25.39	0.00	25.560	25.39	0.68	25.022	25.39	1.45
Average			2.81			1.51			0.34			0.74			2.07
**Discharge Current (mA)**	**EXP (h)**	**KiBaM (h)**	**ERR (%)**	**EXP (h)**	**KiBaM (h)**	**ERR (%)**	**EXP (h)**	**KiBaM (h)**	**ERR (%)**	**EXP (h)**	**KiBaM (h)**	**ERR (%)**	**EXP (h)**	**KiBaM (h)**	**ERR (%)**
20	36.714	36.940	0.62	37.402	36.940	1.24	37.984	36.940	2.75	37.835	36.940	2.37	37.133	36.940	0.52
30	24.749	24.799	0.20	25.087	24.799	1.15	25.385	24.799	2.31	25.560	24.799	2.98	25.022	24.799	0.89
Average			0.41			1.19			2.53			2.67			0.71
**Discharge Current (mA)**	**EXP (h)**	**T-KiBaM ^†^ (h)**	**ERR (%)**	**EXP (h)**	**T-KiBaM ^†^ (h)**	**ERR (%)**	**EXP (h)**	**T-KiBaM ^†^ (h)**	**ERR (%)**	**EXP (h)**	**T-KiBaM ^†^ (h)**	**ERR (%)**	**EXP (h)**	**T-KiBaM ^†^ (h)**	**ERR (%)**
20	36.714	36.711	0.01	37.402	37.398	0.01	37.984	37.978	0.02	37.835	37.828	0.02	37.133	37.133	0.00
30	24.749	24.646	0.42	25.087	25.107	0.08	25.385	25.497	0.44	25.560	25.396	0.64	25.022	24.929	0.37
Average			0.21			0.05			0.23			0.33			0.19
**Discharge Current (mA)**	**EXP (h)**	**T-KiBaM ^‡^ (h)**	**ERR (%)**	**EXP (h)**	**T-KiBaM ^‡^ (h)**	**ERR (%)**	**EXP (h)**	**T-KiBaM ^‡^ (h)**	**ERR (%)**	**EXP (h)**	**T-KiBaM ^‡^ (h)**	**ERR (%)**	**EXP (h)**	**T-KiBaM ^‡^ (h)**	**ERR (%)**
20	36.714	36.866	0.41	37.402	37.361	0.11	37.984	37.815	0.44	37.835	38.061	0.60	37.133	37.271	0.37
30	24.749	24.750	0.00	25.087	25.082	0.02	25.385	25.386	0.01	25.560	25.552	0.03	25.022	25.022	0.00
Average			0.21			0.07			0.23			0.31			0.19

${}^{†}$ Adjust using discharge current equal to 20 mA; ${}^{‡}$ Adjust using discharge current equal to 30 mA.
